# LncRNAs AK058003 and MVIH Overexpression in the Blood Samples of Iranian Breast Cancer Patients

**DOI:** 10.29252/ibj.25.2.93

**Published:** 2020-11-04

**Authors:** Fatemeh Akhavan Attar, Mana Oloomi, Shiva Irani, Masoumeh Azizi, Loabat Geranpayeh

**Affiliations:** 1 Department of Biology, Science and Research Branch, Islamic Azad University, Tehran, Iran;; 2 Department of Molecular Biology, Pasteur Institute of Iran, Tehran, Iran;; 3 Department of Molecular Medicine, Biotechnology Research Center; Pasteur Institute of Iran, Tehran, Iran;; 4 Department of 4th Surgery, Sina Hospital, Tehran, Iran

**Keywords:** Breast cancer, Long noncoding RNA, Real-time PCR

## Abstract

**Background:**

LncRNAs are considered as novel biological regulators and potential cancer biomarkers. LncRNAs MVIH and AK058003 are associated with microvascular invasion in HCC. In BC, upregulated MVIH and AK058003 expression levels have been shown to promote cell proliferation, though LncRNA-AK058003 acts as a tumor suppressor in HCC.

**Methods:**

Blood samples were collected from 30 healthy women and 30 female BC patients. RNA was extracted from the blood of both groups, and cDNA was then synthesized. Real-time PCR was used to measure the expression level of LncRNA-AK058003 and MVIH.

**Results:**

The expression level of two LncRNAs in the blood samples of BC patients increased significantly compared with healthy individuals. The levels of AK058003 and MVIH were not associated with lymph node metastasis (*p* = 0.402 and *p* = 0.39), tumor size (*p* = 0.76 and *p* = 0.461), and TNM stage (*p* = 0.574 and *p* = 0.711), respectively. **Conclusion: **As per our findings, LncRNA-AK058003 could serve as a suitable indicator for low stage of BC. In addition, the increased level of LncRNA-MVIH could be considered as a biomarker for BC, which needs more evaluation in the future.

## INTRODUCTION

Breast cancer is recognized as a high incident cancer and the leading cause of mortality among females, especially in developing countries^[^^1^^,^^2^^]^. In Iran, the incidence of BC is 21.4% among women, and BC accounts for ~25% of the total number of cancers, equivalent to 22.4 people per one million of Iranian women^[^^3^^]^. Therefore, the early detection of BC is essential.

About 70% of Iranian women were diagnosed with a dangerous stage of BC due to the lack of organized screening and shortage of educational programs for the timely diagnosis of BC. Some of the most critical issues in the early diagnosis of BC are hormonal and environmental factors ^[^^4^^]^.

An increasing number of novel treatment strategies have been expanded for BC, such as immunotherapy, gene therapy and targeted molecular therapy. Although satisfactory therapeutic results have been achieved, BC remains the most current cancer among women. 

Non-coding RNAs, a new topic in cell biology, are defined as RNA transcripts that never translated into a protein (so-called ‘non-coding’)^[^^5^^]^. Many LncRNAs act in various pathological and physiological processes, such as differentiation, proliferation, and cellular metabolism^[^^5^^]^. Some of the LncRNAs have also vital roles in regulating oncogenic processes, including metastasis and apoptosis; however, the exact mechanisms of LncRNAs function require further investigation^[^^6^^,^^7^^]^. 

LncRNA-AK058003 has been reported to be upregulated in gastric cancer and supposed to promote cancer metastasis by targeting the *SNCG*^[^^8^^]^. The unregulated LncRNA-AK058003 activates SNCG, which in turn stimulates the proliferation, invasion, and migration of BC^[^^8^^,^^9^^]^. The expression levels of LncRNA-AK058003 have been indicated to be downregulated in HCC tissues. In addition, LncRNA-AK058003 binds human antigen R, as an essential regulator of RNA metabolism, and acts as a precursor of miR-15a in regulating SNCG expression in HCC^[^^10^^]^.

A former study has reported an association between LncRNA-AK094613 and MVIH, and its expression levels can be upregulated in tumor tissues. In addition, LncRNA-MVIH can activate angiogenesis by inhibiting the secretion of phosphoglycerate kinase 1, which is related to glucose metabolism pathway^[^^11^^]^. Another investigation has suggested that MVIH levels enhance in tumor tissues compared to adjacent normal tissues. The increased expression level was significantly correlated with tumor size, TNM stages, and lymph node metastasis. Furthermore, patients with the high levels of MVIH expression had poor prognosis, and the knockdown of MVIH expression by siRNA could inhibit cell proliferation and invasion^[^^12^^]^. 

The aim of this study was to determine the gene expression of the LncRNAs, AK058003 and AK094613 (MVIH), in human blood BC specimens by qRT-PCR and to evaluate their relationship in terms of deregulation and clinical characteristics in Iranian BC patients.

## MATERIALS AND METHODS

**Patients and specimens**

Thirty normal and 30 BC patients were included in this study. The participants were chosen from different Hospitals (Sina, Farmaniyeh, and Moheb Kosar) in Tehran. Fresh blood samples (5 ml) were collected and transferred to Pasteur Institute of Iran for RNA preparation. None of the patients had received preoperative cancer treatments, including radiotherapy or chemotherapy. 

**RNA isolation and cDNA synthesis**

RNA samples were extracted from whole blood using a commercial kit (Jena Bioscience, Germany). The OD A260:A280 ratio and concentration of extracted RNAs were spectrometrically determined (BioTek, USA). The cDNA synthesis was performed using the BioFact kit (Korea) as per manufacturer’s instruction, in which 1 μl of Oligo(dT), 2 μl of RNA (5 µg), 7 μl of double-distilled water, and 10 μl of buffer were combined in a total reaction volume of 20 μl. The mixture was then incubated at 24 °C for 5 min, followed by 50 °C for 30 min, to synthesize the cDNA.

**qRT-PCR** **assay**

The expression levels of LncRNAs were quantified by Eva Green premix (WizPure qPCR Master). According to the protocol, 1 μl of each forward and reverse primer, 2 μl of cDNA, 10 μl of Master mix, and 6 μl of water were mixed for universal analysis of DNA samples. The real-time PCR cycles were run in a Rotor-Gene Q machine (Corbett, Germany). The cycles were set as follows: an initial denaturation at 95 °C for 10 min, followed by 40 cycles of 95 °C for 10 s (denaturation), 60 °C for 15 s (annealing), and 72 °C for 20 s (extension). The melting temperature was 72-95 °C. The sequences of primers used in this study are represented in Table 1, which manufactured by Microsynth, Germany. Each experiment was repeated two times, and *β-actin* was used as an internal control. Forward (5'-CTCTTCCAGCCTTCCTTCCT-3') and reverse (5'-AGCACTCTGTTGGCGTACAG-3') primers of β-actin were applied for the normalization of real-time PCR.

**Statistical analysis**

Data analysis between two groups and the comparison of multiple groups were performed by SPSS 16.0 using student’s *t*-test and the one-way analysis of variance (ANOVA), respectively. The *t*-test also measured the relationship between the clinicopathological characters of patients and LncRNAs expression levels in human BC blood specimens. The LinReg PCR software (version 3, 2015) was applied to calculate the amount of Ct and efficiency for each chart. The relative gene expression level was calculated using the comparative Ct method (^2-^ΔΔct) and the amount of fold change was determined using GraphPad Prism 5.0 (GraphPad Software, La Jolla, CA, USA). To draw a heatmap diagram, online software (CIMminer, One Matrix, NIH) was used. All the information was represented as mean ± SD. *p* values less than 0.05 were considered statistically significant. 

**Table1:** Primers used in this study for real-time PCR

**LncRNA**	**Forward**	**Reverse**	**Length**
*MVIH*	GAGACAGGATTTAGCCGTGTTG	AGCACTTTGGAAGGCTTAGACA	84
*AK058003*	GGGAACAAAGATGGTTTCTACGT	ACTGGTTCATAGTTAGGCTGGAT	199

**Ethical statement**

The above-mentioned sampling protocols were approved by the Research Ethics Committee of Pasteur Institute of Iran, Tehran, Iran (ethical code: IR.PII.REC.1397.008). Written informed consents were provided by all the participants. 

## RESULTS

The expression levels of LncRNAs in the blood of 30 BC patients and 30 healthy women were analyzed. The frequency of AK058003 and MVIH expression level and their correlation with the clinicopathological information of the patients are shown in Table 2. 

**Fig. 1:**
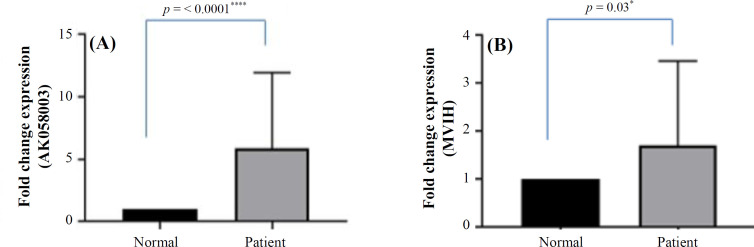
The relative expression level of LncRNAs AK058003 and MVIH in the blood samples of 30 BC patients. LncRNA-AK058003 (A) and LncRNA-MVIH (B) expressions were significantly higher in BC compared with normal blood samples. Also, the expression levels of MVIH and AK058003 were observed to be significant. ΔCt values were used to measure the gene expression, which was normalized by β-actin expression levels

**Fig. 2:**
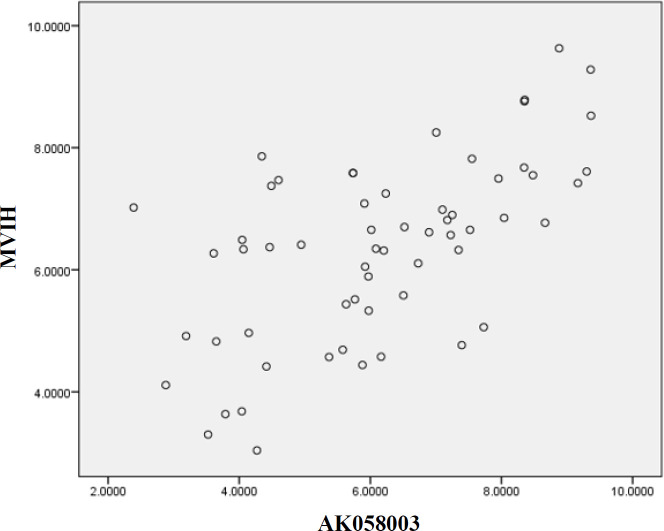
The relative expression level of LncRNA-MVIH and LncRNA-AK058003 (Pearson correlation = 0.609^**^ and Spearman's Rrho = 0.599^**^).

**Fig. 3:**
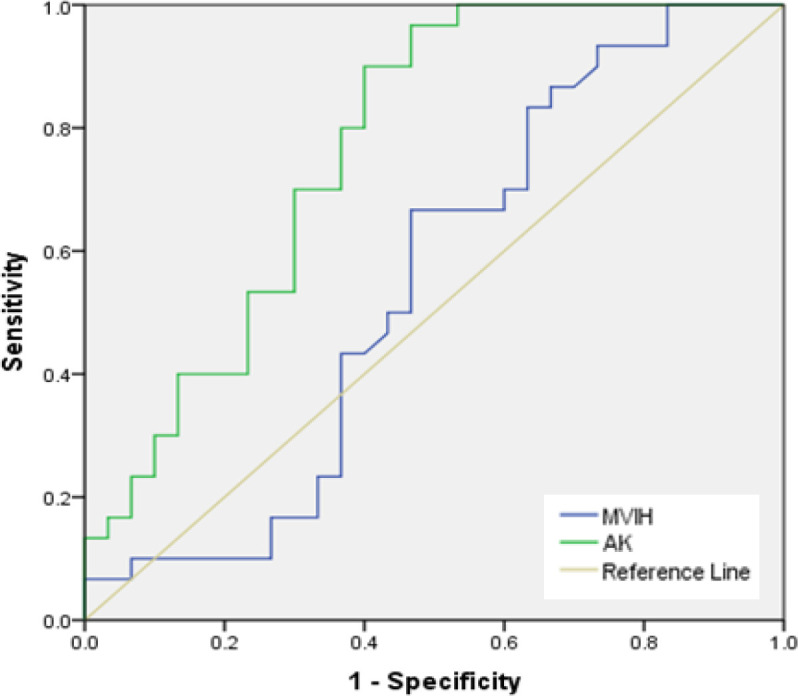
ROC curve showing the sensitivity and specificity of LncRNAs AK058003 and MVIH

Based on qRT-PCR analysis, AK058003 and MVIH expression levels were upregulated in BC samples (Fig. 1). The expression level of AK058003 and MVIH before and after normalization, calculated by LinReg PCR software, indicated a significant increase in the expression levels of both LncRNAs in BC blood specimens. In Figure 2, the relative expression level correlation of LncRNAs MVIH and AK058003 were calculated by Pearson and Spearman's method. To determine a suitable biomarker for cancer detection, rock marker (ROC curve) was also drawn in Figure 3, suggesting that AK058003 is more sensitive marker (*p* < 0.001), but MVIH is a marker with more specificity.

**Fig. 4:**
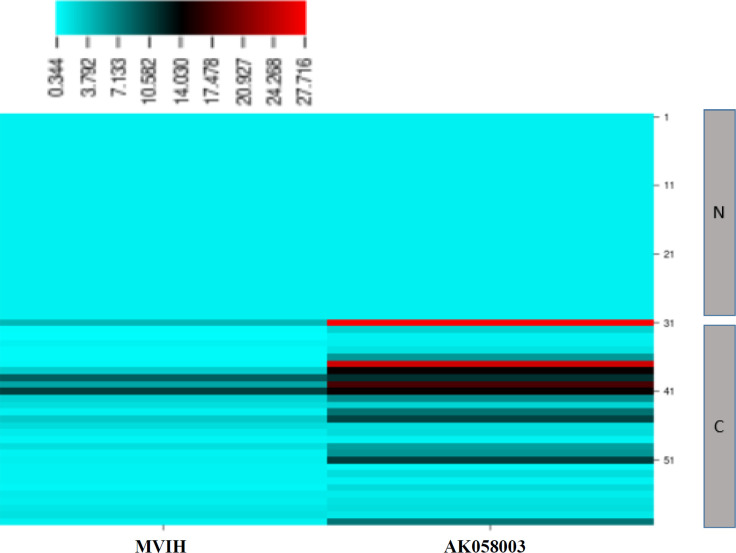
Heatmap diagram of LncRNAs AK058003 and MVIH in normal and BC patients. N, normal; C, breast cancer patients

In the heatmap diagram, the expression levels of LncRNAs AK058003 and MVIH in normal and BC patients are depicted in each sample (Fig. 4). The diagram also displays the expression levels of LncRNAs MVIH and AK058003 in each patient comparing the normal expression level and shows that AK058003 LncRNA expression is significantly higher in normal women.

## DISCUSSION

Previous investigations have shown that LncRNAs play a crucial role in cancer organization and development. Further studies started to concentrate on the regulatory function of LncRNAs in cancer^[^^13^^-^^18^^]^. The expression levels of LncRNAs may facilitate novel drug targets and help to find diagnostic biomarkers for BC^[^^18^^]^. Cai *et al.*^[^^19^^]^ have demonstrated that the colon cancer-associated transcript 2 was considerably overexpressed in BC cell lines and BC tissues, and it was related to clinical prognostic factors. Zhen-Lin *et al.*^[^^20^^]^ have also observed a significantly enhanced expression levels of Z38 in BC tissues compared with adjacent healthy tissues. This information denotes that Z38 is upregulated in BC tissues and may induce tumorigenesis of BC cells^.^

**Table 2:** Clinicopathologic characteristics of patients and expression level of LncRNAs AK058003 and MVIH

**Clinicopathological ** **factors**	**Frequency**	**AK058003**		***p *** **value**	**Frequency**	**MVIH**		***p*** **value**
	**AK058003**	**Low**	**High**		**MVIH**	**Low**	**High**	
Age (year)								0.37
<50	53.4	2	14	0.15	53.4	6	24	
>50	46.6	1	13		46.6	6	24	
								
Size (cm)				0.76				0.461
<2.5	60	2	17		60	8	22	
>2.5	40	1	10		40	5	25	
								
Type				0.557				0.557
Invasive ductal carcinoma	94	1	28		94	8	22	
Invasive lobular carcinoma	6	2			6	2	0	
								
Lymph nodes metastasis				0.402				0.39
Positive	46.6	1	13		46.6	3	27	
Negative	53.4	2	14		53.4	9	21	
								
TNM stage				0.574				0.711
1	47	1	13		47	7	23	
2	23	1	6		23	1	29	
3	30	1	8		30	2	27	
								
Diffrentiataion grade				0.829				0.624
G1	26	1	7		26	6	24	
G2	53	2	14		53	5	25	
G3	21		6		21	1	29	

LncRNA-H19 is one of the oncogenic LncRNAs that its expression has widely been investigated in blood samples^[^^21^^]^^. ^The expression levels of H19 in cancer biopsy specimens and plasma increased dramatically, which was significantly associated with the expression of estrogen and progesterone receptors and lymph node metastasis^[^^22^^]^. However, Xiaoqin *et al.*^[^^10^^]^ have indicated that LncRNA-AK058003 acts as a tumor suppressor, inhibiting HCC cell growth and metastasis *in vitro *and *in vivo*^.^ LncRNA-AK058003 is a transcript with 1,197 nucleotides and located on the reverse strand of the chromosome 10q22^[^^23^^]^^.^ Lei *et al.*^[^^1^^]^ have disclosed that the upregulation of MVIH in BC tissues, which were higher than adjacent noncancerous tissues, was correlated with Ki67 expression. Kumarswamy *et al.*^[^^24^^]^ have reported that circulating LncRNAs were from the tissues, while the expression of MVIH and AK058003 were detected in blood. The results showed that the levels of MVIH and AK058003 in BC were much higher than their levels in the controls. Due to the existence of RNases, circulating LncRNAs are thought to be unstable^[^^25^^]^. In the present study, we assessed the MVIH and AK058003 levels in blood. Our result confirmed that circulating LncRNAs were stable, and we, for the first time, detected the expression of MVIH and AK058003 in blood for further analysis. Shin *et al.*^[^^26^^]^ measured the expression level of LncRNA-NEAT1 in the blood specimens of BC patient by qRT-PCR. Zhen-Lin *et al.*^[^^20^^]^ applied GAPDH and Liu *et al.*^[^^27^^]^ applied β-actin as the internal controls for qRT-PCR. We used both GAPDH and β-actin as controls, though β-actin showed more satisfactory result. 

In this study, the ROC curves showed that AK058003 is more sensitive marker (*p* < 0.001) than MVIH for BC diagnosis. However, one limitation of the study was the sample size that was very limited, and our findings required to be validated with more cases. Herein, the correlations between LncRNAs (MVIH and AK058003) and between their expression level and different clinicopathological features of BC patients were analyzed. Our findings revealed that the high expression of LncRNAs AK058003 and MVIH 

were not related to stages, based on TNM, differentiation grade, and lymph node metastasis. However, He *et al.*^[^^23^^]^ found a close link between the expression of AK058003 and lymph node metastasis. This difference may correlate with the number of patients, though they are in low stage of BC.

In conclusion, the present study provides insights into the expression levels of AK058003 in relation to MVIH in the blood specimens of BC patients. For the first time in this study, the expression of LncRNAs AK058003 and MVIH demonstrated to be significantly higher in BC patients in comparison to controls. Further studies are needed to fully understand the molecular mechanisms of LncRNA application for diagnostic purposes in future.
